# Primary Structure Analysis of Antifungal Peptides from Cultivated and Wild Cereals

**DOI:** 10.3390/plants7030074

**Published:** 2018-09-12

**Authors:** Eugene Rogozhin, Dmitry Ryazantsev, Alexey Smirnov, Sergey Zavriev

**Affiliations:** 1 Shemyakin and Ovchinnikov Institute of Bioorganic Chemistry, Russian Academy of Sciences, ul. Miklukho-Maklaya 16/10, 117997 Moscow, Russia; d.yu.ryazantsev@gmail.com (D.R.); szavriev@ibch.ru (S.Z.); 2 Gause Institute of New Antibiotics, ul. Bolshaya Pirogovskaya, 11, 119021 Moscow, Russia; 3 Department of Plant Protection Timiryazev Russian Agricultural University, ul. Timiryazevskaya 49, 127550 Moscow, Russia; smirnov@timacad.ru

**Keywords:** antimicrobial peptides, wild and cultivated cereals, primary structure analysis, biological activity

## Abstract

Cereal-derived bioactive peptides with antimicrobial activity have been poorly explored compared to those from dicotyledonous plants. Furthermore, there are a few reports addressing the structural differences between antimicrobial peptides (AMPs) from cultivated and wild cereals, which may shed light on significant varieties in the range and level of their antimicrobial activity. We performed a primary structure analysis of some antimicrobial peptides from wild and cultivated cereals to find out the features that are associated with the much higher antimicrobial resistance characteristic of wild plants. In this review, we identified and analyzed the main parameters determining significant antifungal activity. They relate to a high variability level in the sequences of C-terminal fragments and a high content of hydrophobic amino acid residues in the biologically active defensins in wild cereals, in contrast to AMPs from cultivated forms that usually exhibit weak, if any, activity. We analyzed the similarity of various physicochemical parameters between thionins and defensins. The presence of a high divergence on a fixed part of any polypeptide that is close to defensins could be a determining factor. For all of the currently known hevein-like peptides of cereals, we can say that the determining factor in this regard is the structure of the chitin-binding domain, and in particular, amino acid residues that are not directly involved in intermolecular interaction with chitin. The analysis of amino acid sequences of alpha-hairpinins (hairpin-like peptides) demonstrated much higher antifungal activity and more specificity of the peptides from wild cereals compared with those from wheat and corn, which may be associated with the presence of a mini cluster of positively charged amino acid residues. In addition, at least one hydrophobic residue may be responsible for binding to the components of fungal cell membranes.

## 1. Introduction

Defense peptides, including antimicrobial peptides, are essential components of plant innate immunity and occur in all of the described species from the botanical families of flowering plants. Numerous research papers devoted to antimicrobial proteins and peptides from gymnosperm plants have demonstrated the so-called evolutionary succession and antiquity of these defense molecules in the plant kingdom. The overwhelming majority of wild flowering species (dicotyledonous and monocotyledonous) grow in unfavorable conditions (e.g., sunlight, availability of mineral sources, soil fertility, optimal acidity, etc.). At the molecular level, this usually results in allelopathic interactions between plants cohabitants [1,2]. For example, more “aggressive” wild plant species can be more successful through the synthesis of metabolites suppressing the growth and development of other plant species. These substances can be actively secreted by the root system to soil or by aerial vegetative parts (leaves, stems, and flowers).

High resistance to biotic and abiotic environmental stress factors is vital to plants. Many of these plants (in particular, cereals) grow in high-salinity soils [3,4]. For instance, lyme grass (*Leymus arenarius*) is an endemic species that is widespread in the northern parts of Russia, and lives in acidic soils along the coastlines of the White and Barents seas, including littoral areas with low mean air temperatures [5,6]. Elongated couch grass (*Elytrigia elongata*) is resistant to extremely high concentrations of organic and inorganic salts in soil, but grows in alkaline soils with high air temperatures [7,8]. In some cases, there are little or no data about the influence of some biotic stress factor on wild plants, which include microorganism infections and damage by pests, nematodes, or other invertebrates. The reason is a lack of economic relevance in agriculture. A few exceptions are the wild relatives of cultivated plants, such as cereals and leguminous breeds and hybrids, which may potentially be used to create novel breeds with high resistance to diseases.

Disease agents and pests that have economic significance for several cultivated cereals (soft and durum wheat, rye, barley, rice, etc.) are under intensive study. Methods to suppress pathogenecity and related damage have been developed and optimized based on a combination of agrotechnical, chemical, and biological approaches. Defense polypeptides are present in all types of flowering plants, but differences may occur in some plant organs at different stages of plant ontogenesis. In this work, a comparative structural analysis of antimicrobial peptides (AMPs) was conducted. AMPs were qualitatively and quantitatively characterized in the kernels of wild and cultivated cereals to identify any differences in their amino acid sequences [9,10,11,12]. The study purpose was to gain further understanding of the contribution of this direction of innate immunity to the greater tolerance of wild plants compared to that of cultivated plants.

Defensins, hevein-like peptides, thionins, and alpha-hairpinins (hairpin-like peptides) were investigated. We found that these AMPs were involved in the defense reaction of innate and adapted plant immunity. Thus, the defensin and thionin families are “pathogenesis-related proteins” (PR-proteins) [13,14]. For hevein-like and hairpin-like peptides, a reliable increase in the transcription level of their encoding genes in response to abiotic or biotic stress factors was identified by semi-quantitative PCR amplification [15,16].

## 2. Comparative Analysis of the Primary Structure of Defensins Isolated from Wild and Cultivated Cereals

Defensins are defense peptides isolated from numerous angiosperm plants [17,18,19,20,21,22,23,24,25,26,27], *Ginkgoaceae* [28], and some coniferous species (*Pinophyta*) [29]. Initially, defensins isolated from wheat and barley kernels were assigned to the thionin subfamily (γ-thionins) [30,31]; however, they were demonstrated to have a homology with defensins from mammals and insects, which was the reason to consider these defensins as a new family [31,32].

Plant defensins are the most highly expressed defense molecules in plant immunity. As in animal immunity, they can be functionally activated in response to any phytopathogenic microorganisms (fungi, bacteria, oomycetes, etc.). These molecules typically have a rather wide spectrum of biological activity, although their main function is the interaction with the cell membrane of a target cell, followed by permeabilization, disruption of the transmembrane electrochemical potential, deprivation of essential metabolites, and, finally, cell death [33,34,35].

There are defensins whose number is limited to one or two per cereal species (wheat, rye, barley, sorghum, and millet). Thus, we investigated the peptidomic composition of hexaploid wheat (*Triticum kiharae*) kernels using protein chemistry techniques. We also isolated and structurally characterized two defensin subfamilies. The first group was the well-known gamma-thionin family (originally D-defensins). The second group was the “omega-type” family that is characterized by a novel structural motif differing from the traditional cysteine-stabilized αβ-motif by the position of cysteine residues in the polypeptide chain [36]. AMPs from this group were identified in multigenomic wheat species and wild cereals [37].

Barnyard grass (*Echinochloa crusgalli*) is well-characterized in terms of its defense peptide composition. Using a combination of acidic extraction and several types of liquid chromatography, two high homologous defensins (Ec-AMP-D1 and Ec-AMP-D2) with a single amino acid substitution were isolated (Ala46His) [32]. Notably, a similar “pair” of defensins containing single substitution was discovered in several dicotyledonous plant species. The two most notable features are the presence of a variable amino acid substitution in the loop fragment of molecules, which is critical for the spatial orientation, and a quantitative level of the antimicrobial activity. Both defensins were tested against a broad spectrum of plant pathogenic fungi from *Fusarium* (*F. graminearum, F. oxysporum, F. verticillioides*), *Bipolaris sorokiniana*, *Phoma betae*, *Botrytis cinerea*, some specific pathogens of corn (*Zea mays*) (*Colletotrichum graminicola*, *Diplodia maydis*), and oomycetes (*Pythium debarianum* and *Phytophthora infestans*) at concentrations ranging from 1.7 µM to 20 µM. In all of the cases, the D1 molecule was more active than D2. Importantly, these peptides could cause the morphological destruction of conidia and mycelium in oomycetes, but not in fungi [38].

On the basis of multiple alignments of well-known amino acid sequences of defensins, we may conclude that the primary structure homology is typical of cultivated and wild species (usually, 55–70% of homology). However, the key factor determining the significant antimicrobial activity (primarily antifungal) of wild cereals is related to a high variability of C-terminal fragments and a high percentage of hydrophobic amino acid residues in their biologically active defensins.

**Figure 1:**
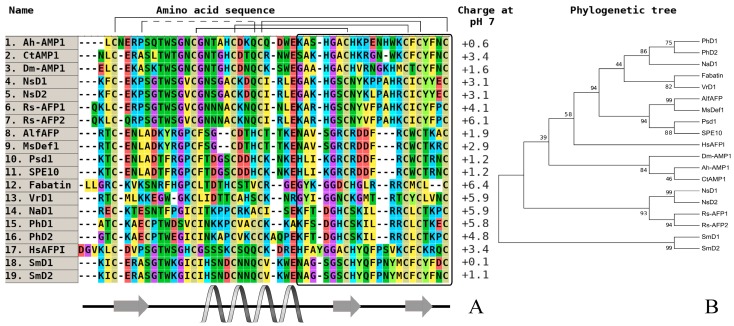
The alignment (**A**) and phylogenetic tree (**B**) of defensins from dicots. Аmino acids are highlighted in color, and сonserved disulfide bonds are connected by black lines. The variable С-end is marked with a black rectangle. Conservative elements of the secondary structure are shown under the alignment in accordance with RsAFP1 from radish (*Raphanus sativus*) seeds [29]. Ah-AMP1—defensin from *Aesculus hippocastanum* (GenBank ID: AAB34970); CtAMP1—defensin from *Clitoria ternatea* (GenBank ID: Q7M1F2); DmAMP1—defensin from *Dahlia merckii* (GenBank ID: P0C8Y4); NsD1—defensin D1 from *Nigella sativa* (UniProt ID: P86972); NsD2—defensin D2 from *N. sativa* (UniProt ID: P86973); Sm-D1—defensin D1 from *Stellaria media* (GenBank ID: C0HL82); Sm-D2—defensin D2 from *S. media* (GenBank ID: C0HL83); Rs-AFP1—antifungal protein 1 from *Raphanus sativus* (GenBank ID: AAB22709); Rs-AFP2—antifungal protein 2 from *R. sativus* (GenBank ID: AAB22710); AlfAFP—antifungal peptide from *Medicago sativa* (GenBank ID: AAG40321); MsDef1—defensin 1 from *M. sativa* (GenBank ID: AAV85433); Psd1—defensin 1 from *Pisum sativum* (UniProt ID: P81929); SPE10—defensin from *Pachyrhizus erosus* (GenBank ID: AAT80338); Fabatin—defensin from *P. erosus* (GenBank ID: ACI02057); VrD1—defensin from *Vigna radiata* (GenBank ID: AAR08912); NaD1—defensin from Nicotiana alata (GenBank ID: Q8GTM0); PhD1—defensin 1 from *Petunia hybrida* (GenBank ID: Q8H6Q1); PhD2—defensin 2 from *P. hybrida* (GenBank ID: Q8H6Q0); Hs-AFP1—defensin 1 from *Heuchera sanguinea* (GenBank ID: AAB34974).

**Figure 2:**
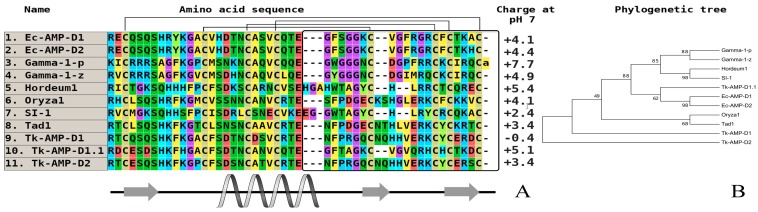
The alignment (**A**) and phylogenetic tree (**B**) of defensins from wild and cultivated cereals (monocots). Аmino acids are highlighted in color, and сonserved disulfide bonds are connected by black lines. The variable С-end is marked with a black rectangle. Conservative elements of the secondary structure are shown under the alignment according to gamma-purothionin from soft wheat (*T. aestivum*) (Gamma-1-p) (CP) [40]. Ec-AMP-D1—defensin 1 from *Echinochloa crusgalli* (UniProt ID: P86518) (WP); Ec-AMP-D2—defensin 2 from *E. crusgalli* (UniProt ID: P86519) (WP); Gamma-1-p—defensin (Gamma-1-purothionin) from *Triticum aestivum* (UniProt ID: P20158) (CP); Gamma-1-z—defensin (Gamma-zeathionin-1) from *Zea mays* (UniProt ID: P81008) (CP); Oryza1—defensin from *Oryza sativa* Japonica Group (GenBank ID: BAD23741) (CP); Tad1—defensin (Gamma-thionin) from *T. aestivum* (GenBank ID: BAC10287) (CP); Tk-AMP-D1—defensin 1 from *Triticum kiharae* (UniProt ID: P84963) (CP); Tk-AMP-D2—defensin 2 from *T. kiharae* (UniProt ID: P84968) (CP); Tk-AMP-D1.1—defensin 1.1 from *T. kiharae* (UniProt ID: P84965) (CP). All structures are marked: CP—cultivated plant species, WP—wild plant species.

AMPs from cultivated forms usually exhibit weak or no activity. Plant defensins from dicots and monocots are cationic amphiphilic polypeptides, and their positive charge is primarily localized in the N-terminal part of a molecule, and initiates the interaction with negatively charged components of bacteria and cell walls of fungi and oomycetes (Figure 1 and Figure 2, the plant defensin sequences from the literature performed UniProt/SwissProt algorithms to generate the alignments via CLUSTAL OMEGA interactive service (https://www.ebi.ac.uk/Tools/msa/clustalo/). As illustrated on the phylogenetic tree, all of the molecules from seven different branches, most of them combining purely very high homologous sequences, as a rule, are isolated from one plant species. To date, some defensins from cultivated cereals (wheat, barley, sorghum, and corn), which are a unique group of molecules participating in plant immunity, have been studied, but these have exhibited a low level of antimicrobial activity (20 µM to 100 µM) [33]. Defensins from cereal barnyard grass (*E. crusgalli*) from the *Poaceae* family also, in contrast to previously studied wild plants, demonstrate significant antifungal activity against plant pathogenic micromycetes and oomycetes [39]. It is typical, as they are localized on various branches on phylogenetic tree (Figure 2), but this correlation is not expressed, which is similar to the defensins from dicots.

The discovered differences are a striking example of the biological activity preservation of defensins from wild plant forms to achieve suitability, competitive ability, and adaptation in biocenoses, which were previously lost in the breeding process. Concerning defensins from other plant species, highly homologous AMPs (RsAFP1 and RsAFP2) differing by two amino acid substitutions were isolated from radish (*Raphanus sativus* L.) seeds [39,40]. These substitutions were localized in the β1-helical and α-helical regions, respectively, and led to the accumulation of a higher total positive charge in the Rs-AFP2 peptide compared to that in Rs-AFP1, and to higher antimicrobial activity against a model fungal species, *Fusarium culmorum* [40]. At the same time, the site-directed mutagenesis of Rs-AFP2 based on the addition of arginine residues led to an increase in the antifungal activity of a mutant molecule relative to its native form. These results are in accordance with the data obtained for Ec-AMP-D1/D2 [38].

It should be noted that so far, some defensins of cultivated cereals (wheat, barley, sorghum, and corn) have been studied. Defensins are the unique group of molecules that are involved in the protection of these plants from fungal diseases [41]. The results obtained in our investigations confirm the existing literature data on the study of grain defensins [42], and show that wheat defensins (*T. kiharae*) (synthetic hexaploid, which is obtained by crossing the wild species of *Triticum timopheevii* and *Aegilops squarrosa* aegilops) have an insignificant antifungal activity against several phytopathogenic filament fungi. We show in this review that defensins isolated from wild-growing plant–barnyard grass (*E. crusgalli*), which also belongs to the Cereals family (*Poaceae*), in contrast to the previously studied wild forms of plants, show significant antifungal activity against a number of phytopathogenic fungi and oomycetes. The detected differences are a good example of the preservation of antifungal activity in the defensins of wild cereals for the achievement of fitness, competitiveness, and adaptation in biocenoses; those capabilities are mostly lost in the process of cultivation. The detection of antifungal activity of *Echinochloa* defensins opens new possibilities for the analysis of structural–functional relationships, including in particular the identification of residues, which are determinants of biological activity. Basically, this can be achieved in three ways: by comparing the amino acid sequences of highly homologous plant peptides of different phylogenetic remoteness or from closely related plants that are highly contrasting in biological (antifungal) activity; by conducting a site-directed mutagenesis of structures of active peptides; and by the construction of chimeric molecules combining different parts of peptide structures differing in their activity (strong or weak).

It should be also noted that due to a wide spread of massively parallel transcriptome sequencing in the last years, a lot of genes encoding defensin-like peptides have been discovered in many plants, including cereals [43,44,45,46,47]. These genes are able to be expressed after the creation of biologically active molecules on different stages of plant ontogeny in normal or stress conditions.

## 3. Investigation of Structural Determinants of Other AMPs (Thionins, Hevein-Like Peptides, and Alpha-Hairpinins), Which Provide Higher Antifungal Activity to Wild Cereals

Thionins are structurally similar to plant defensins. A principal difference between them is the presence of a single alpha-helix as an element of the secondary structure in defensins. They contain a cluster of two antiparallel alpha-helices that are coupled by a beta-turn, and are localized in vacuoles [48].

Although thionins are the first discovered and described AMP family from plants, the number of isolated and characterized thionins is lower compared to that in the defensin family [49,50]. The basic members in cereals and all other plants are the alpha and beta-purothionins isolated from the kernels of soft wheat (*Triticum aestivum*). Their three-dimensional structures were determined using X-ray diffraction [51,52,53,54]. Subsequently, thionins from other cultivated cereals were isolated and characterized in detail (hordothionins from barley (*Hordeum vulgare*), zeathionins from corn (*Zea mays*), and avesins from rice (*Oryza sativa*)) [24,55,56,57]. An interesting peculiarity of thionins is a higher total positive charge at neutral pH as well as significant membrane-active features that can impart cytotoxic effects toward some tumor cell lines in vitro. Their influence on the expression level of oncogenes, tumor suppressor genes [58,59,60], and ability to bind DNA can also decrease the toxic effect of heavy metal ions [61]. These properties are also found in thionins from wild dicots [62,63,64,65,66]. These molecules belong to the eight-cysteine thionin subfamily. There is another subfamily with six-cysteine type thionins isolated from white mistletoe (*Viscum album*), which are called viscotoxins [67,68]. The antimicrobial activity of these peptides was determined against Gram-positive and Gram-negative bacteria, yeasts, fungi, and oomycetes at an IC_50_ concentration of 1–15 µg/mL (Table 1) [69]. It is typical that there is quite determined divergence between the eight and six-cysteine-containing thionins, which is expressed their localization in different branches of the built phylogenetic tree.

**Figure 3:**
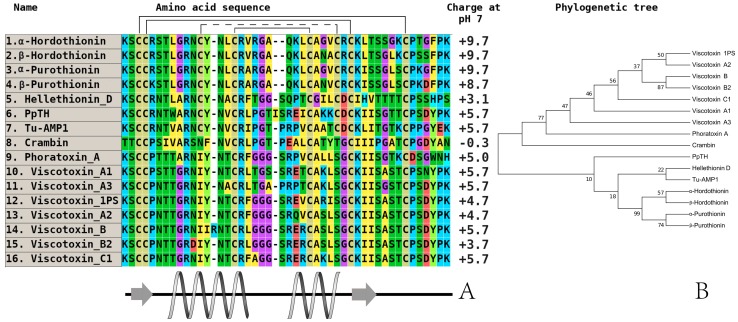
The alignment (**A**) and phylogenetic tree (**B**) of plant thionins from eight and six-cysteine type subfamilies. Аmino acids are highlighted in color, and сonserved disulfide bonds are connected by black lines. Conservative elements of the secondary structure are shown under the alignment according to alpha-purothionin from soft wheat (*T. aestivum*), (CP) [57]. α-hordothionin—thionin from Hordeum vulgare (monocotyledons, UniProt ID: P01545), (CP); β-hordothionin—thionin from *H. vulgare* (UniProt ID: P21742), (CP); α-purothionin—thionin from *Triticum aestivum* (monocotyledons, GenBank ID: AFQ60540), (CP); β-purothionin—thionin from *T. aestivum* (AAB71137), (CP); hellethionin_D—thionin from *Helleborus purpurascens* (dicotyledons, UniProt ID: P60057), (WP); PpTH—thionin from *Pyrularia pubera* (dicotyledons, UniProt ID: P07504), (WP); Tu-AMP1—thionin from *Tulipa gesneriana*, (monocotyledons, [22]), (CP); crambin—thionin from *Crambe hispanica* subsp. *abyssinica* (dicotyledons, UniProt ID: P01542), (WP); phoratoxin_A—thionin from *Phoradendron leucarpum* subsp. *tomentosum* (dicotyledons, UniProt ID: P01539), (CP); viscotoxin_A1—thionin from *Viscum album* (dicotyledons, GenBank ID: 3C8P_B), (WP); viscotoxin_A3—thionin from *V. album* (GenBank ID: VTVAA3), (WP); viscotoxin_1PS—thionin from *V. album* (UniProt ID: P01537), (WP); viscotoxin_A2—thionin from *V. album* (UniProt ID: P32880), (WP); viscotoxin_B—thionin from *V. album* (UniProt ID: P08943), (WP); viscotoxin_B2—thionin from *V. album* (UniProt ID: P08943), (WP); viscotoxin_C1—thionin from *V. album* (UniProt ID: P83554), (WP). All structures are marked: CP—cultivated plant species, WP—wild plant species.

A comparative analysis of amino acid sequences isolated from the main species of wild cereals did not achieve positive results. For example, we could not predict any structure–function relationship between members of this family in cultivated cereals or wild plants. However, if we consider the similarity of some estimated physicochemical parameters (charge, localization of secondary structure elements) between thionins (Figure 3) and defensins (Figure 1 and Figure 2), the presence of high divergence on a fixed part of any polypeptide (e.g., C-terminus) may be a key factor.

Hevein-like AMPs possess structural homology with hevein, the first real chitin-binding peptide isolated from *Hevea brasiliensis* [70]. Apart from hevein, lectins, chitinases from I/IV classes, and hevein-like AMPs belong to chitin-binding polypeptides [71,72].

**Figure 4:**
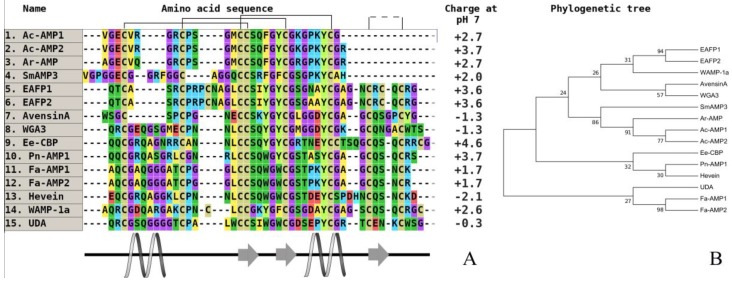
The alignment (**A**) and phylogenetic tree (**B**) of plant hevein-like peptides from six and 10-cysteine type subfamilies. Аmino acids are highlighted in color, and conserved disulfide bonds are connected by black lines. Conservative elements of the secondary structure are shown under the alignment according to WAMP-1a from wheat (*T. kiharae*), (CP) [68]. Ac-AMP1—antimicrobial peptide 1 from *Amaranthus caudatus* (dicotyledons, GenBank ID: AAB22103), (CP); Ac-AMP2—antimicrobial peptide 2 from *A. caudatus* (dicotyledons, GenBank ID: AAB22102), (CP); Ar-AMP—antimicrobial peptide from *A. retroflexus* (dicotyledons, GenBank ID: Q5I2B2), (WP); SmAMP3—antimicrobial peptide 3 from *Stellaria media* (dicotyledons, GenBank ID: C0HJU5), (WP); EAFP1—antifungal peptide 1 from *Eucommia ulmoides* (dicotyledons, UniProt ID: P83596), (WP); EAFP2—antifungal peptide 2 from *E. ulmoides* (dicotyledons, UniProt ID: P83597), (WP); WGA3—agglutinin isolectin 3 from *T. aestivum* (monocotyledons, UniProt ID: P10969), (CP); Ee-CBP—hevein-type antimicrobial peptide from *Euonymus europaeus* (dicotyledons, (CP); Pn-AMP1—antimicrobial peptide from *Ipomoea nil* (dicotyledons, UniProt ID: P81591), (WP); Fa-AMP1—antimicrobial peptide from *Fagopyrum esculentum* (dicotyledons, UniProt ID: P0DKH7), (CP); Fa-AMP2—antimicrobial peptide from *F. esculentum* (dicotyledons, UniProt ID: P0DKH8), (CP); hevein—hevein from *Hevea brasiliensis* (dicotyledons, GenBank ID: AAA33357), (CP); WAMP-1a—antimicrobial peptide from *T. kiharae* (monocotyledons, PDB ID: 2LB7_A), (CP); UDA—agglutinin from *Urtica dioica* (dicotyledons, UniProt ID: P11218), (WP). All structures are marked: CP—cultivated plant species, WP—wild plant species.

The real revelation occurred when a novel structural type of hevein-like antimicrobial peptides with a 10-cysteine motif was isolated from the kernels of wheat (*T. kiharae*). Curiously, only for cultivated cereals were these peptides obtained first. It allowed a reconsideration of the modern classification of hevein AMPs to diverge them into two subfamilies: six-cysteine-containing peptides, such as peptides from amaranth (*Amaranthus caudatus, A. retroflexus*) [73,74,75], common chickweed (*Stellaria media*) [76,77], and 10-cysteine-containing peptides [78,79]. The most famous examples are wheat antimicrobial peptides (WAMPs) from *T. kiharae* and lyme grass antimicrobial peptides (LAMP) from *L. arenarius* families [80,81]. To date, in addition to wheat, a similar homologue was also isolated and characterized from the wild cereal lyme grass (*L. arenarius*) (Figure 4) [81].

These AMPs are highly homologous, but a single amino acid substitution in the chitin-binding domain is critical for the level of antifungal activity, which does not correlate with fungalysin inhibition [82,83]. High antifungal activity was detected in all of the studied hevein-like plant peptides, but it was preliminary found in dicots. Their antimicrobial spectrum is sufficiently broad and includes the inhibition of filamentous and yeast-like microorganisms at a mean concentration of 10 µg/mL [84]. The WAMP-1a peptide can affect both Gram-positive and Gram-negative bacteria. After a more detailed examination of the data on the degree of antifungal activity in vitro (Table 2) for all of the currently known hevein-like peptides of cereals, we can say that the determining factor in this regard is the structure of the chitin-binding domain, in particular, amino acid residues, not directly involved in intermolecular interaction with the polymer.

As we noted earlier, the only amino acid variable replacement in the structure of the WAMP family peptides has a significant effect on their functionality, which is also confirmed by comparison with the amino acid sequence of the antimicrobial peptide LAMP-1a from wild-grown plant lyme grass (*L. arenarius*). Additionally, the presence of the last arginine residue in the WAMP sequence compensates for a positive charge in the C-terminal fragment of the molecule and increases the binding to chitin. Accordingly, it is not possible to draw conclusions about the differences in the level of activity between the peptides of cultivated and wild cereals based on the example of peptides from this family (including a small size of sample).

The members of both subfamilies typically have a long chitin-binding domain that functions at the initial stage of interaction with chitin from fungal cell walls. For example, one of these mechanisms is most likely responsible for the ability of WAMP peptides to inhibit the hydrolytic activity of the zinc metalloproteinase fungalysin (that is produced and secreted by the plant pathogenic fungus *Fusarium verticillioides* in infection) and reduce the inactivation of the catalytic domain of corn chitinase IV type [85]. There is not enough information about the structural diversity of peptides from this family to understand the differences between their contribution to the different resistances of wild and cultivated cereals. The data indicate that the high diversity of homologous genes encoding these molecules among a wide range of species from the *Poaceae* family (wild and cultivated) may broaden their ability to be activated under the action of signal molecules and heavy metals in plants [86].

Hairpin-like peptides (alpha-hairpinins) are a family of defense molecules of plant immunity, which were found and described relatively recently. They include short alpha-helical peptides with four cysteine residues that form two disulfide bridges, generating a beta-hairpin between alpha helices. The four-cysteine maize basic peptide (MBP-1) isolated from *Z. mays* kernels in 1992 was the first described member of these polypeptides. It exhibited high antimicrobial activity against some fungi, specific corn pathogens, and Gram-negative bacteria [87]. Peptides with an analogous structure were detected in dicotyledonous plants: nut (*Macadamia integrifolia*), buckwheat (*Fagopyrum esculentum*), winterweed (*Veronica hederifolia*), pumpkin (*Cucurbita maxima*), loofah (*Luffa aegyptiaca*), and chickweed (*S. media*) [16,88,89,90,91,92,93]. Two of them were found to be trypsin inhibitors (VhTI peptide from winterweed (*V. herdefolia*) and BWI-2c from buckwheat (*F. esculentum*)) [92,93]. The spatial structure of alpha-hairpinins’ molecular complex with trypsin was determined by X-ray diffraction [92]. For the first time in wild cereals since the description of MBP-1, a novel family of antimicrobial hairpin-like peptides (EcAMPs) was discovered in barnyard grass (*E. crusgalli*) [94,95,96], as well as in the sequel that was also in cultivated wheat (*T. kiharae*) [97]. Studying the biological activity mechanisms of these peptides at the cellular level allowed us to conclude about their fungistatic influence on microscopic fungi, which was implemented as a delay in the spore germination and growth power of hyphae [94,98]. The data on the quantitative antifungal activity of some hairpin-like peptides are presented in Table 3.

The data presented demonstrates that isolated AMPs influence different fungus species in a broad range of active concentrations. It is characteristic that according to the results of comparative testing of biological activity in vitro, in general, certain experimentally found quantitative levels of antifungal effect of alpha-harpinins isolated from grain crops are less expressed than the level of their homologues from wild species, including dicots.

These results may be explained by some fungi being able to cause diseases in many plant species, including cereals, whereas some other filamentous fungus are represented as having a too-narrow specificity toward cereals.

**Figure 5:**
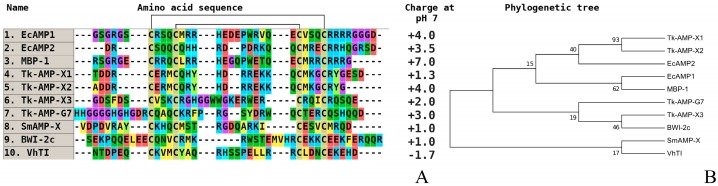
The alignment (**A**) and phylogenetic tree (**B**) of plant hairpin-like peptides. Аmino acids are highlighted in color, and conserved disulfide bonds are connected by black lines. EcAMP1—antimicrobial peptide 1 from *E. crusgalli* (monocotyledons, GenBank ID: B3EWR6); EcAMP2—antimicrobial peptide 2 from *E. crusgalli* (GenBank ID: B3EWR6); MBP-1—antimicrobial peptide from *Z. mays* (monocotyledons, GenBank ID: AAB23306); Tk-AMP-X1—antimicrobial peptide X1 from *T. kiharae* (monocotyledons, [88]); Tk-AMP-X2—antimicrobial peptide X2 from *T. kiharae* [97]; Tk-AMP-X3—antimicrobial peptide X3 from *T. kiharae* [97]; Tk-AMP-G7—antimicrobial peptide G7 from *T. kiharae* [97]; SmAMP-X–antimicrobial peptide from *S. media* (dicotyledons, GenBank ID: U4N938); BWI-2c—trypsin inhibitor 2c from *F. esculentum* (dicotyledons, UniProt ID: P86794); VhTI—trypsin inhibitor from *Veronica hederifolia* (dicotyledons, UniProt ID: P85981).

The multiple alignment of amino acid sequences of alpha-hairpinins isolated from plant organs, including cultivated and wild cereal species, reveals a stable low percentage of homology between them. The only exception is a pair of MBP-1 and EcAMP1 from corn and barnyard grass, which are highly homologous. Our experiments and the literature data allowed us to identify a fragment of the polypeptide chain of plant alpha-hairpinins that is critical for their biological activity. This is a secondary structure element, a beta-hairpin, that connects two alpha-helices and is about 10–13 amino acid residues in length (Figure 5).

Based on the detailed analysis of amino acid sequences of hairpin fragments in alpha-hairpinins, we may draw conclusions about the higher antifungal activity and greater specificity of peptides from wild cereals compared to peptides from wheat and corn. These parameters may result from the presence of a mini cluster of positively charged amino acid residues (for effective interaction with negatively charged carbohydrate components of the superficial layer of fungal cell walls and any polymers, such as beta-1,3-glucans) [99]. Concerning the localization of the hairpin-like peptides from cereals and dicotyledonous plants, we can see that it also repeats the low homology of the molecules based on primary structure analysis. So, EcAMP1/2 is not similar to Tk-AMP-X1/2; rather, it is opposed to them, as they really contrast on antifungal activity.

Furthermore, at least one hydrophobic residue (e.g., tryptophan) is responsible for binding to components of fungal cell membranes (e.g., sphingolipids or ergosterols) [34,35].

## 4. Conclusions

This review for the first time provides a comparative analysis of the primary structures of AMPs from the most represented families of cultural and wild cereals’ seeds. These peptides were analyzed to identify differences in their amino acid sequences. Due those differences, significant variations in the level of antimicrobial activity in relation to both the pathogens of specific fungal diseases of cereals, and pathogens with a wide range of host plants, are observed. For this analysis, we selected AMPs that belong to both the most studied families (defensins, thionins, and hevein-like peptides), and the poorly studied families (alpha-hairpinins, of hairpin-like peptides). As a result of the analysis, a large rate of variability of C-terminal fragments for peptides from the defensin family was detected. It should be noted that for representatives of wild forms of cereals, the presence of hydrophobic amino acid residues in those fragments is more intrinsic. We analyzed the similarity of various physicochemical parameters between thionins and defensins. The presence of highly pronounced divergence on a fixed part of any polypeptide that is close to defensins could be a determining factor. For all of the currently known hevein-like peptides of cereals, we can say that the determining factor in this regard is the structure of the chitin-binding domain, and in particular, amino acid residues that are not directly involved in intermolecular interaction with the polymer. The analysis of amino acid sequences of alpha-hairpinins revealed that the more significant quantitative antifungal activity and wide specificity of the peptides from wild cereals compared to those in the peptides from wheat and corn may be associated with parameters such as the presence of a mini cluster of positively charged amino acid residues and at least one hydrophobic residue that is responsible for binding to the components of fungal cell membranes. The conclusion indicates the possibility of using highly active AMPs of wild species of cereals as a basis for the creation of transgenic cultivated plants (including cereals) that express genes, coding AMPs. In addition, the application of genome-editing techniques for the promoters of target genes, coding potentially active AMPs in cultural cereals, may significantly increase their expression, which can also lead to a decrease in susceptibility to diseases.

## Figures and Tables

**Table 1:** Antifungal activity of some known thionins isolated from cereals (IC_50_, µM).

Peptide/Microbe	α 1 -Purothionin	α -Hordothionin	γ -1-H-Hordothionin	γ -1-P-Purothionin	γ -1-Zeathionin	Tk-AMP-BP1	Tk-AMP-BP2
*Bipolaris sorokiniana*	3.2	5.0	Not tested	Not tested	Not tested	5.6	32.0
*Botrytis cinerea*	Not tested	20.0	Not tested	Not tested	Not tested	Not tested	32.0
*Fusarium oxysporum*	3.9	5.0	4.0	7.6	7.0	6.0	Not tested
*F. solani*	Not tested	5.0	Not tested	Not tested	Not tested	Not tested	Not tested
*F. verticillioides*	1.9	2.7	2.2	3.5	3.0	4.5	Not tested
*Neurospora crassa*	Not tested	10.0	Not tested	Not tested	Not tested	Not tested	Not tested

**Table 2:** Antifungal activity of some known hevein-like peptides from cereals (IC_50_, µM).

Peptide/Microbe	WAMP1a (+R)	WAMP1b (−R)	WAMP2a (A34K)	WAMP3a (A34E)	WAMP4a (A34N)	LAMP-1a	Ar-AMP
***Bipolaris sorokiniana***	3.2	5.0	Not tested	Not tested	Not tested	5.6	32.0
***Botrytis cinerea***	Not tested	20.0	Not tested	Not tested	Not tested	Not tested	32.0
***Fusarium oxysporum***	3.9	5.0	4.0	7.6	7.0	6.0	Not tested
***F. solani***	Not tested	5.0	Not tested	Not tested	Not tested	Not tested	Not tested
***F. verticillioides***	1.9	2.7	2.2	3.5	3.0	4.5	Not tested
***Neurospora crassa***	Not tested	10.0	Not tested	Not tested	Not tested	Not tested	Not tested

**Table 3:** Antifungal activity of some known alpha-hairpinins (IC_50_, µM).

Peptide/Microbe	EcAMP1	EcAMP2	EcAMP3	Tk-AMP-X1	Tk-AMP-X2	MBP-1	Sm-AMP-X
***Alternaria alternata***	16.0	>32.0	19.8	Not tested	28.8	Not tested	14.8
***Aspergillus niger***	>32.0	>32.0	22.4	Not tested	>32.0	Not tested	4.0
***B. sorokiniana***	18.2	>32.0	15.0	Not tested	Not tested	Not tested	>32.0
***C. graminicola***	>10	Not tested	Not tested	>30.0	>30.0	Not tested	Not tested
***D. maydis***	>10	Not tested	Not tested	30.0	17.0	Not tested	Not tested
***F. graminearum***	4.5	>32.0	5.5	7.5	7.5	4.0	Not tested
***F. oxysporum***	8.8	>32.0	9.6	Not tested	13.5	Not tested	6.8
***F. solani***	4.0	>32.0	4.8	Not tested	8.5	Not tested	8.0
***F. verticillioides***	8.1	>32.0	5.2	15.0	10.0	Not tested	Not tested
***P. infestans***	16.3	>32.0	14.0	Not tested	25.4	Not tested	>32.0
***P. ultimum***	14.4	Not tested	Not tested	Not tested	Not tested	Not tested	>32.0
